# Biotransformation of perfumery terpenoids, (−)-ambrox® by a fungal culture *Macrophomina phaseolina* and a plant cell suspension culture of *Peganum harmala*

**DOI:** 10.1186/1752-153X-6-82

**Published:** 2012-08-05

**Authors:** Syed Ghulam Musharraf, Sheeba Naz, Asma Najeeb, Saifullah Khan, M Iqbal Choudhary

**Affiliations:** 1 H.E.J. Research Institute of Chemistry, International Center for Chemical and Biological Sciences, University of Karachi, Karachi 75270, Pakistan; 2 Department of Chemistry, College of Science, King Saud University, Riyadh, 1145, Saudi Arabia

**Keywords:** (−)-ambrox, Macrophomina phaseolina, Peganum harmala, Biotransformation

## Abstract

**Background:**

Biotransformation offers chemo enzymatic system to modify the compounds into their novel analogues which are difficult to synthesize by chemical methods. This paper describes the biotransformational studies of ambrox, one of the most important components of natural Ambergris (wale sperm) with fungal and plant cell culture.

**Results:**

Biotransformation of (−)-ambrox (**1**) with a fungal cell culture of *Macrophomina phaseolina* and a plant cell suspension cultures of *Peganum harmala* yielded oxygenated products, 3*β*-hydroxyambrox (**2**), 6*β*-hydroxyambrox (**3**), 1*α*-hydroxy-3oxoambrox (**4**), 1*α*,3*β*-dihydroxyambrox (**5**), 13,14,15,16-tetranorlabdane-3-oxo-8,12-diol (**6**), 3-oxoambrox (**7**), 2*α*-hydroxyambrox (**8**), 3*β*-hydroxysclareolide (**9**), and 2*α*,3*β*-dihydroxyambrox (**10**). Metabolite **4** was found to be new compound. These metabolites were structurally characterized on the basis of spectroscopic studies.

**Conclusion:**

Nine oxygenated metabolites of (−)-ambrox (**1**) were obtained from *Macrophomina phaseolina* and *Peganum harmala.* Enzymatic system of screened organisms introduced hydroxyl and keto functionalities at various positions of compound **1** in a stereo- and regio-controlled manner.

## Background

Fungal cultures have been widely used for the structural modification of compounds to afford structurally novel derivatives [1,2], while utilization of plant cell suspension cultures for structural transformations is comparatively a new approach [3]. Only limited numbers of natural products have been transformed through different plant cell cultures. This paper describes the biotransformation of (−)-ambrox (**1**) by using cell suspension culture of plant *Peganum harmala,* and a fungal cell culture of *Macrophomina phaseolina*.

*Peganum harmala* L. (Harmal, Zygophyllaceae) is a bushy herb which is widely distributed in the Europe, Africa and Asia. *P. harmala* has been used in traditional system of medicine. Cell cultures of *P. harmala* have been extensively employed used to produce *β*-carboline alkaloids which exhibit interesting biological activities, such as enzyme inhibition, anticancer, antioxidant, immunomodulatory and antileishmanial activity [4,5]. *P. harmala* has also been used for the bioconversion of terpenes [6,7]. It also has the potential to discriminate between enantiomeric pairs of substrates [8].

(−)-Ambrox (**1**) is a highly fragrant constituent of Ambergris, a metabolite of the sperm whale. During drifting in the sea for many years, Ambergris is oxidatively decomposed by the action of sea water, air and sunlight yields several odorous compounds [8,9]. Among these compounds, (−)-ambrox (**1**) has a strong amber-like odor and graded as good as “Civet” and “Musk” [10]. (−)-Ambrox (**1**) is much stronger perfume than the (+)-ambrox. In recent years, it has become more difficult to obtain various kinds of animal perfumes because of the gradual reduction in the world’s wild resources, and efforts to conserve wild animals. However, (−)-ambrox has been synthesized from various precursors including sclareol [11], cyclization of polyprenoids [12] and labdanolic acid [13].

In continuation of our biotransformational studies on bioactive compounds [14-20], structural transformation of (−)-ambrox (**1**) with *Macrophomina phaseolina* and *Peganum harmala* was studied which afforded nine oxidative metabolites **210**. Compounds **2**, **3**, **5**, **610** were previously reported as metabolites of compound **1** [18,19,21].

## Experimental

### General experimental conditions

(−)-Ambrox® (**1**) (the term “ambrox” is trademark registered in the name of the Swiss company, Firmenich SA) was purchased from Sigma-Aldrich (USA). The melting points were determined on Buchi 535 melting point apparatus and were uncorrected. JASCO DIP–360 Digital polarimeter was used to measure the optical rotations in chloroform. FTIR-8900 Spectrophotometer was used to record IR spectra in CHCl_3_. The ^1^ H-NMR spectra were recorded on 500 MHz, while ^13^ C-NMR spectra were recorded on Bruker AMX-500 operating at 125 MHz using CDCl_3_ as solvent. Chemical shifts were reported in *δ* (ppm), relative to SiMe_4_ as internal standard, and coupling constants (*J*) were measured in Hz. The EI-MS and HRFAB-MS were measured on Jeol HX 110 mass spectrometer. TLC was performed on Si gel precoated plates (PF_*254*_, 20 × 20, 0.25 mm, Merck, Germany). Ceric sulphate spraying reagent was used for the staining of compounds on TLC. All reagents used were of analytical grades.

### Biotransformation of 1 by plant cell culture

Seeds of *Peganum harmala* were collected from the campus of International Center for Chemical and Biological Sciences (ICCBS), University of Karachi. A voucher specimen (#74168) of plant *Peganum harmala*, from which the seeds were collected, was deposited in the Herbarium of Department of Botany, University of Karachi. Seeds were germinated in 300 mL jars, each having 25 mL of Murashige and Skoog (MS) media supplemented with 3% sucrose. The calli was initiated from germinated young leaves, cultivated in 300 mL jars each having 25 mL of MS medium containing 0.1 mg/L 6-benzylaminopurine (BAP), 1 mg/L α-naphthalene acetic acid (NAA) solidified by agar (6 g/L) at 25°C ± 1°C (under complete darkness). The cultures were maintained on the same medium at 25°C ± 1°C (in the dark by sub culturing after every four weeks). Two-week-old friable calli were used to initiate cell suspension culture in the above mentioned MS medium without agar (solidifying agent). The cells were grown on shaking at 110 rpm for 15 days at 25°C under a 16 h photoperiod.

After 15 days, the cells were harvested and introduced onto the above mentioned freshly prepared medium. After 4 days of transfer to fresh media, the substrate **1** (600 mg) was dissolved in DMSO (10 mL) and evenly distributed among 10 culture flasks (1 liter) having 400 mL cell suspension media under aseptic conditions, which were kept for 15 days under the same conditions. During the cell growth period, aliquots from culture were taken out every day and analyzed by TLC in order to determine the degree of transformation of substrate. In all experiments, one control flask without cell culture (for checking substrate stability), and another flask with cell culture but without exogenous substrate (for checking endogenous metabolite), were used.

### Biotransformation of compound 1 with fungal cell cultures

Stock culture of the fungus was stored on Sabouraud dextrose agar at 4°C prior to use. The media for *Macrophomina phaseolina* (KUCC 730, Karachi University Culture Collection, Department of Botany) were prepared by adding the following chemicals into distilled H_2_O (2.0 L): glucose (20.0 g), glycerol (20.0 mL), peptone (10.0 g), yeast extract (10.0 g), KH_2_PO_4_ (10.0 g), and NaCl (10.0 g). The fermentation medium thus obtained was adjusted to pH 7.0 and distributed among 20 flasks of 250 mL capacity (100 mL in each), and autoclaved. Compound **1** was dissolved in acetone. The resulting clear solution was evenly distributed among 20 flasks (20 mg/0.5 mL in each flask), containing 24-h-old stage II cultures, and fermentation was continued for further 12-days on a rotatory shaker (100 rpm) at room temperature. In all experiments, one control flask without biomass (for checking substrate stability), and one flask without exogenous substrate (for the identification of endogenous metabolites) were used.

### Extraction and isolation of metabolites

In case of fungal cell culture*,* fermentation experiment was allowed to process for four days after the final feed. The culture media and mycelium were separated by filtration. The mycelium was washed with CH_2_Cl_2_ (1 L) and the filtrate was extracted with CH_2_Cl_2_ (3 × 2 L). The combined organic extract was dried over anhydrous Na_2_SO_4_, and evaporated under reduced pressures to obtain a brown gum (2.62 g), which was purified by using column chromatography. Elution with gradient mixture of ethyl acetate in petroleum ether afforded compounds **2** (11 mg, 19% ethyl acetate in petroleum ether), **3** (7 mg, 23% ethyl acetate in petroleum ether), **4** (21 mg, 31% ethyl acetate in petroleum ether), **5** (16 mg, 37% ethyl acetate in petroleum ether) and **6** (24 mg, 41% ethyl acetate in petroleum ether). In case of plant cell culture, the similar procedure was used as described above. From 600 mg of compound **1**, CH_2_Cl_2_ extract (1.86 g) was obtained from the fermentation broth after 6 days, and subjected to column chromatography over silica gel with gradient elution of petroleum ether-ethyl acetate to obtain compounds **7** (19 mg, petroleum ether-EtOAc, 79: 21), **8** (15 mg, petroleum ether-EtOAc, 59: 41), **9** (21 mg, petroleum ether-EtOAc, 56: 44) and **10** (18 mg, petroleum ether-EtOAc, 56: 44).

*1α-Hydroxy-3oxoambrox (****4****)*

White crystalline solid; mp 124–125°C; [α]_D_^25^ = −27.° (*c* 0.5, MeOH); IR (CHCl_3_) *ν*_max_ 3383, 2927, 2884, 2361, 1702, 1455 cm^-1^; ^1^ H NMR (CDCl_3_, 500 MHz): Table 1; ^13^ C NMR (CDCl_3_, 125 MHz): Table 1; EI-MS *m/z* 266 (2, M^+^), 251 (100, [M -Me]^+^), 233 (6, [M-Me-H_2_O] ^+^), 207 (3), 167 (8) 149 (5), 139 (8), 123 (7), 113 (8), 95 (10), 85 (19). HREI-MS *m/z* 266.1864 (C_16_H_26_O_3_; calc. 266.1882).

**Table 1:** ^**1**^ **H- and**^**13**^ **C-NMR data of metabolite 4 in CDCl**_**3**_

**Position**	** 4**		
	***δ***_**H**_**(mult., *J *in Hz)**^**#**^	***δ***_**C**_^**¤**^	**Mult.**
1	4.53 brs	70.1	CH
2	2.23 m, 1.65 m	48.0	CH_2_
3	-	218.2	C
4	-	46.8	C
5	1.35 m	58.0	CH
6	1.71 m, 1.51 m	34.1	CH_2_
7	1.75 m, 1.48 m	41.1	CH_2_
8	-	79.2	C
9	1.42 m	60.3	CH
10	-	35.7	C
11	1.69 m, 1.51 m	22.9	CH_2_
12	3.96 dt (3.3, 9.0), 3.81 dd (8.1, 16.5)	64.4	CH_2_
13	1.36 s	21.9	CH_3_
14	1.39 s	14.9	CH_3_
15	1.17 s	24.7	CH_3_
16	1.41 s	23.4	CH_3_

^# 1^ H NMR data, 500 MHz.

^¤13^ C NMR data, 125 MHz.

### Time-course study and TLC densitometry analysis

Time course experiment was conducted during the fermentation, one culture flask was harvested daily, extracted with the 300 mL of CH_2_Cl_2_ organic solvent and analyzed by TLC-densitometry method in order to determine the degree of transformation of the substrate. A CAMAG TLC autosampler (Linomat 5) was used for the spotting. Video densitometry of the chromatoplate was carried out with the help of CAMAG Reprostar 3 and the integrated software of WinCATS (Version 1.4.4.6337) was used for the analysis. Precoated silica gel aluminum sheets 60 F-254 (20 cm × 10 cm) were used for the application of samples which were spotted as bands of width 6 mm with a CAMAG 100 μL syringe. Linear ascending development of spotted TLC sheet was carried out in 20 cm × 10 cm twin trough vertical glass chamber (CAMAG) with 10 mL mobile phase (hexane: acetone = 6:4, *v/v*) in unsaturated condition. Developed plate was stained with ceric sulphate. Densitometric scanning was performed in the reflectance-absorbance mode at λ_max_ 550 nm. Picture of TLC plate was obtained by using CAMAG Reprostar 3 with cabinet cover and mounted on digital camera on white R mode.

## Results and discussion

**Figure 1:**
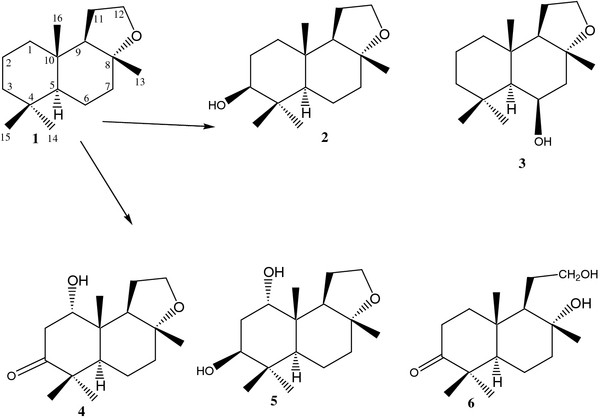
**Biotransformation of compound 1 by a fungal culture of *Macrophomina phaseolina.***

**Figure 2:**
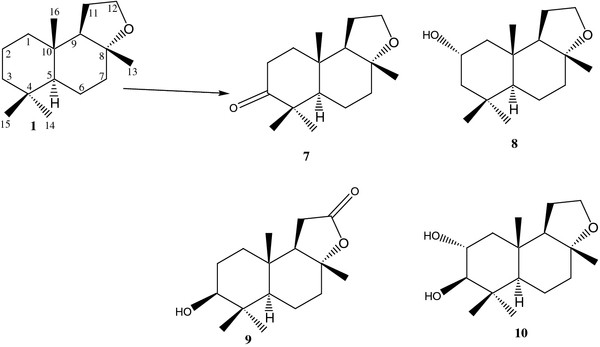
**Biotransformation of compound 1 by a plant cell culture of *Peganum harmala.***

Incubation of compound **1** with fungal cell culture of *Macrophomina phaseolina* for 4 days yielded six oxidative metabolites **2**–**6** (Figure 1), while with *Peganum harmala* (plant cell culture), metabolites **7**–**10** were obtained (Figure 2). Structures of known metabolites (**2**, **3**, **5–10**) were elucidated through comparison of their reported data [18,19,21], while the structure of new metabolite **4** was elucidated through spectroscopic studies and with the comparison of **1**.

**Figure 3:**
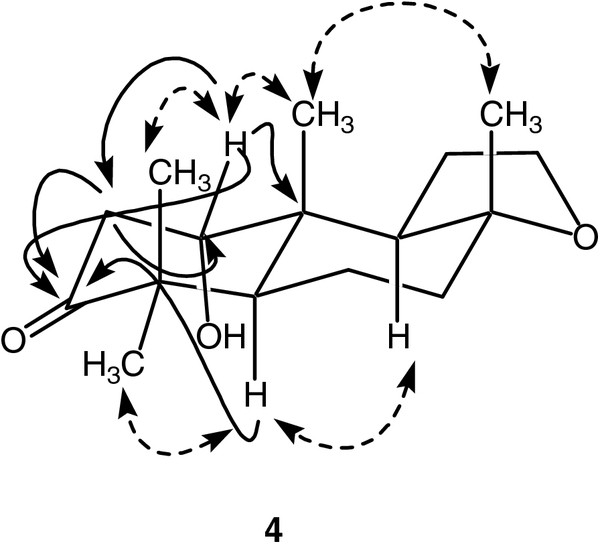
**Key NOESY****and HMBC****interactions in compound 4.**

The HREI-MS of **4** showed the M^+^ at *m/z* 266.1864 (C_16_H_26_O_3_, calc. 266.1882), indicated two new oxygen functionalities in the molecule as compared to substrate **1**. The IR absorption at 3383 cm^-1^ has further indicated the presence of OH group, while absorption at 1702 cm^-1^ indicated the presence of carbonyl group. The ^1^ H NMR spectra displayed an additional methine signal at δ 4.53 (brs), while H-3 signal was missing in comparison to **1**. Moreover, H_2_-11 were appeared at 3.96 (dt, *J* = 9.0 Hz, *J* = 3.3 Hz) and 3.81 (dd, *J* = 16.5 Hz, *J* = 8.0 Hz), similar as in compound **1**. The ^13^ C NMR spectrum of **4** showed two additional downfield signals at δ 70.1 (C-l), and 218.0 (C-3). The new downfield methine proton at δ 4.53 showed couplings with C-2 methylene protons (δ 1.95, 1.52) in COSY-45° spectrum and thus indicated a hydroxylation at C-1. The HMBC spectrum of **4** (Figure 3) showed interactions between H_2_-2 (δ 2.23, 1.65)/C-3 (δ 218.0), C-1 (δ 70.1); H-1 (δ 4.53)/C-2 (δ 48.0), C-3 (δ 218.0), C-10 (δ 35.7); and H_2_-12 (δ 3.96, 3.81)/C-l1 (δ 22.9) and C-9 (δ 60.3), which further supported the proposed structure. The 1α-configuration of the newly introduced –OH was deduced on the basis of NOESY correlations (Figure 3) between Me-16*β* (δ 1.41) and H-1*β* (δ 4.53). The structure of metabolite **4** was identified as 1*α*-hydroxy-3oxoambrox (**4**).

**Figure 4:**
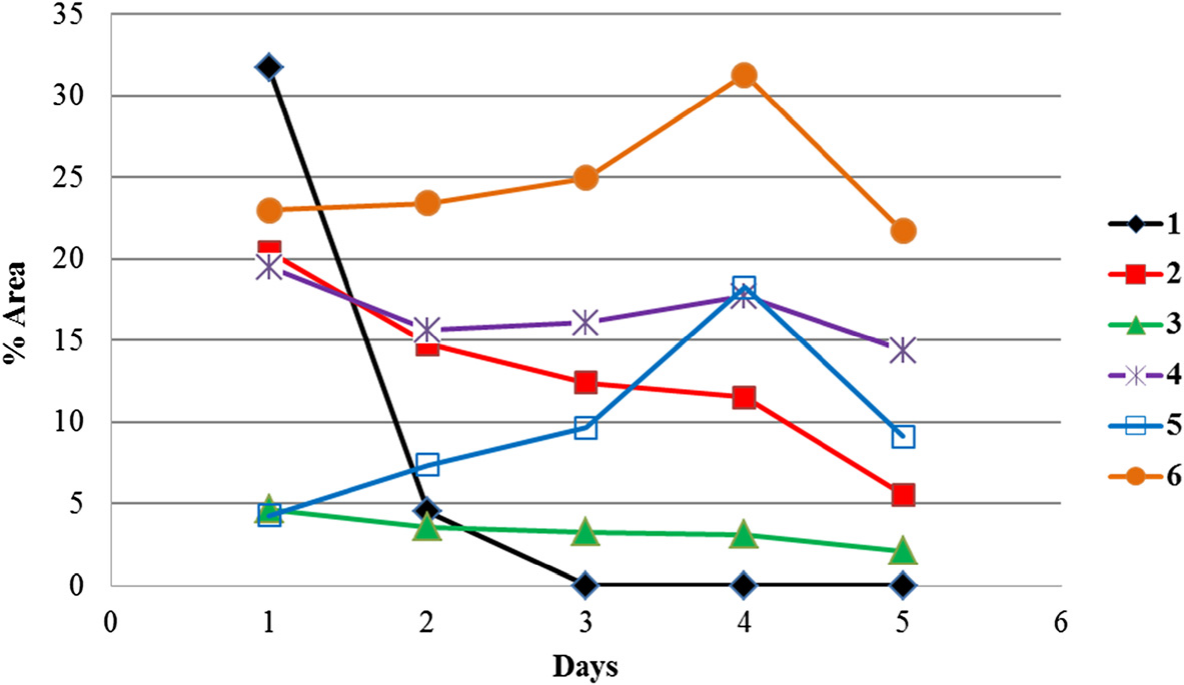
**Time course study of metabolites formation of (−)-ambrox (1) incubated with *Macrophomina phaseolina.***

Time course study was conducted for both the experiments. TLC-densitometric analysis of daily extracted flasks, incubated with *Macrophomina phaseolina*, showed metabolites formation after 24 hrs of incubation, while further continuation of fermentation showed significant changes (Additional file 1: Figure S1). Formation of metabolites **2**, **3**, **4** and **6** were observed after 24 hrs of incubation, while daily analysis of extract on TLC indicated that metabolite **5** appeared in a significant amount after 3-days of treatment, while concentration of compound **2** was gradually decreasing. Further continuation of fermentation causes a decrease in the concentrations of all metabolites (Figure 4), therefore, all flasks were harvested after four days of incubation. Above observation indicated that metabolite **5** arose by the selective oxidation of C-3 hydroxyl group from compound **4**, while metabolite **6** was formed by the oxidation of C-3 hydroxyl group, followed by hydrolysis of ether (compound **2**).

Time course experiment of **1** with *Peganum harmala* indicated that the metabolites **7**–**10** were formed after six days of incubation, while further continuation of fermentation experiments have not caused any significant changes.

In conclusion, resulting biotransformed products represent interesting structural transformations. In case of *Macrophomina phaseolina*, compound **1** gone through an hydroxylation at C-3*β*, C-6*β*, and C-1*α* and regio controlled keto formation at C-3. Moreover ether hydrolysis was also observed. Compound **1** incubated with *Peganum harmala* resulted the production of hydroxylated products at C-2*α* and C-3*β*, while compound **9** showed lactone formation in five member ring i.e. sclareolide skeleton, Formation of scelerids skeleton from ambrox is a well known process biosynthetically. These transformations provide accesses to regions of the ambrox skeletons, which are difficult to be functionalized by conventional chemical methods. These products may therefore be useful for the development of new fragrances.

## Competing interests

The authors declare that they have no competing interests.

## Authors’ contributions

SGM: Participated in the experimental design and manuscript writing. SN: Performed the experiments related to plant cell culture. AN: Performed the experiments related to fungal cell culture. SK: involve in the optimization of plant cell culture. MIC: Significantly contributed in the discussion and the manuscript preparation. All authors read and approved the final manuscript.

## Supplementary Material

Additional file 1 **Figure S1.** Vedio densitometry picture of time course study experiment of (-)-ambrox (**1**) with *Macrophomina phaseolina.* Click here for file

## References

[B1] Atta-ur-Rahman Choudhary MI Musharraf SG Atta-ur-Rahman Frontiers in Natural Product Chemistry Microbial transformation of natural products-a tool for the synthesis of novel analogues of bioactive substance 2005 1 Karachi: Bentham Science Publishers 133 147 Volume 1

[B2] De Carvalho CCCR Da Fonseca MMR Biotransformation of terpenes Biotechnol Adv 2006 24 134 142 10.1016/j.biotechadv.2005.08.004 16169182

[B3] Ishihara K Hamada H Hirata T Nakajima N Biotransformation using plant cultured cells J Mol Catal B: Enzym 2003 23 145 170 10.1016/S1381-1177(03)00080-8

[B4] Asghari G Ihsanpour A Akbari A Production of arbutin by biotransformation of hydroquinone using Peganum harmala, Varthemia persica and Pycnocycla spinosa cell suspension cultures Iran J Pharm Sci 2006 2 91 96

[B5] Astulla A Zaima K Matsuno Y Hirasawa Y Ekasari W Widyawaruyanti A Zaini NC Morita H Alkaloids from the seeds of Peganum harmala showing antiplasmodial and vasorelaxant activities J Nat Med 2008 62 470 472 10.1007/s11418-008-0259-7 18523842

[B6] Asghari G Lockwood GB Stereospecific biotransformation of (±) phenylethyl propionate by cell cultures of Peganum harmala L Iran Biomed J 2002 6 43 46

[B7] Asghari G Saidfar G Mahmudi S Biotransformation of aromatic aldehydes by cell cultures of Peganum harmala L. and Silybum marianum (L.) Gaertn Iran J Pharm Res 2004 2 127 130

[B8] Tanimoto H Oritani T Synthesis of (+)-ambrein Tetrahedron 1997 53 3527 3536 10.1016/S0040-4020(97)00103-8

[B9] Mori K Tamura H Triterpenoid total synthesis, I. Synthesis of ambrein and Ambrox® Eur J Org Chem 1990 4 361 368

[B10] Martres P Perfetti P Zahra JP Waegell B Giraudi E Petrzilka M A short and efficient synthesis of (−)-Ambrox® from (−)-sclareol using a ruthenium oxide catalyzed key step Tetrahedron Lett 1993 34 629 632 10.1016/S0040-4039(00)61637-4

[B11] Ishihara K Ishibashi H Yamamoto H Enantio- and diastereoselective stepwise cyclization of polyprenoids induced by chiral and achiral LBAs. A new entry to (−)-Ambrox, (+)-Podocarpa-8, 11, 13-triene Diterpenoids, and (−)-Tetracyclic polyprenoid of sedimentary origin J Am Chem Soc 2002 124 3647 3655 10.1021/ja0124865 11929254

[B12] Bolster MG Jansen BJM de Groot A The synthesis of (−)-Ambrox® starting from labdanolic acid Tetrahedron 2001 57 5657 5662 10.1016/S0040-4020(01)00493-8

[B13] Musharraf SG Najeeb A Ali RA Ali AA Choudhary MI Metabolites of the fungistatic agent 2β-methoxyclovan-9α-ol by Macrophomina phaseolina J Agri Food Chem 2011 59 3234 3238 10.1021/jf104314f 21391597

[B14] Musharraf SG Najeeb A Hareem S Choudhary MI Biotransformation of 5α-hydroxycaryophylla-4(12),8(13)-diene with Cunninghamella elegans and Rhizopus stolonifer Biocatalysis Biotransform 2011 29 141 146 10.3109/10242422.2011.591927

[B15] Musharraf SG Ahmed MA Ali RA Choudhary MI Hydroxylation of (+)-menthol by Macrophomina phaseolina Biocatalysis Biotransform 2011 29 77 82 10.3109/10242422.2011.577209

[B16] Musharraf SG Najeeb A Khan S Pervez M Ali RA Choudhary MI Microbial transformation of 5α-hydroxycaryophylla-4(12),8(13)-diene with Macrophomina phaseolina J Mol Catal B Enzymatic 2010 66 156 160 10.1016/j.molcatb.2010.04.011

[B17] Nasib A Musharraf SG Hussain S Khan S Anjum S Ali S Rahman A Choudhary MI Biotransformation of (−)-ambrox by cell suspension cultures of Actinidia deliciosa J Nat Prod 2006 69 957 959 10.1021/np050221o 16792418

[B18] Choudhary MI Musharraf SG Sami A Atta-ur-Rahman Microbial transformation of sesquiterpenes, (−)-ambrox® and sclareolide Helv Chim Acta 2004 87 2685 2694 10.1002/hlca.200490238

[B19] Choudhary MI Siddiqui ZA Khan S Saifullah Musharraf SG Atta-ur-Rahman Biotransformation of caryophyllene oxide by cell suspension culture of Catharanthus roseus Z Naturforsch B 2006 61 197 200

[B20] Hashimoto T Noma Y Asakawa Y Biotransformation of terpenoids from the crude drugs and animal origin by microorganisms Heterocycles 2001 54 529 559 10.3987/REV-00-SR(I)7

