# Supplementary material for: Biotransformation of perfumery terpenoids, (−)-ambrox® by a fungal culture Macrophomina phaseolina and a plant cell suspension culture of Peganum harmala
Source: Chem Cent J. 2012 Aug 5;6:82. doi: 10.1186/1752-153X-6-82 (PMC3551650; doi:10.1186/1752-153X-6-82)
Supplement: Additional file 1 — Figure S1. Vedio densitometry picture of time course study experiment of (-)-ambrox (1) with Macrophomina phaseolina. [file 1752-153X-6-82-S1.doc]

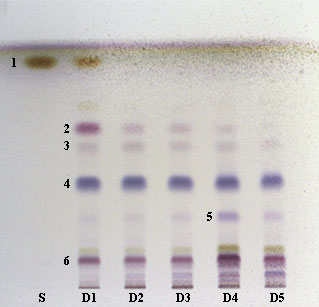


**Supplementary Figure 1.** Vedio densitometry picture of time course study experiment of (-)-ambrox (**1**) with *Macrophomina phaseolina*.
